# Tetraspanins as Potential Therapeutic Candidates for Targeting Flaviviruses

**DOI:** 10.3389/fimmu.2021.630571

**Published:** 2021-04-21

**Authors:** Waqas Ahmed, Girish Neelakanta, Hameeda Sultana

**Affiliations:** ^1^ Department of Biological Sciences, Old Dominion University, Norfolk, VA, United States; ^2^ Center for Molecular Medicine, Old Dominion University, Norfolk, VA, United States

**Keywords:** tetraspanins, arthropods, flaviviruses, exosomes, transmission, ncRNAs

## Abstract

Tetraspanin family of proteins participates in numerous fundamental signaling pathways involved in viral transmission, virus-specific immunity, and virus-mediated vesicular trafficking. Studies in the identification of novel therapeutic candidates and strategies to target West Nile virus, dengue and Zika viruses are highly warranted due to the failure in development of vaccines. Recent evidences have shown that the widely distributed tetraspanin proteins may provide a platform for the development of novel therapeutic approaches. In this review, we discuss the diversified and important functions of tetraspanins in exosome/extracellular vesicle biology, virus-host interactions, virus-mediated vesicular trafficking, modulation of immune mechanism(s), and their possible role(s) in host antiviral defense mechanism(s) through interactions with noncoding RNAs. We also highlight the role of tetraspanins in the development of novel therapeutics to target arthropod-borne flaviviral diseases.

## Introduction

Membrane receptor proteins actively participate in order to control specific cellular functions due to their unique architecture, composition, stability and their ability to regulate the signaling from inside out (1, 2). Tetraspanin proteins containing transmembrane domains are responsible for characteristic functions in membrane compartmentalization and in biological processes including adhesion, viral infection/transmission, differentiation, cell signaling, and motility (2). Several studies have shown that tetraspanins play a significant role in modulation and in trafficking of other host membrane proteins (3–5). Tetraspanins have also been shown to play key role(s) in flaviviruses entry and exit process (2), transmission from arthropod to mammalian cells (6), and in cell-to-cell spreading (7). Arthropod-borne flaviviruses are emerging and re-emerging pathogens responsible for substantial morbidity and mortality with clinical conditions, such as congenital malformations, neurological complications, brain encephalitis, severe hemorrhagic syndromes and multiple organ failures (8, 9). Among viruses that belong to the family of Flaviviridae, the *Flavivirus* genus includes more than seventy small, positive-sense single-stranded RNA viruses transmitted by vectors, such as ticks and mosquitoes (10, 11). This genus comprises of broadly distributed and important human pathogens including dengue virus (DENV), yellow fever virus (YFV), West Nile virus (WNV), Japanese encephalitis virus (JEV), Zika virus (ZIKV), tick-borne: encephalitis virus (TBEV), Langat virus (LGTV), Powassan virus (POWV) and Murray Valley encephalitis virus (MVE) (8, 9, 12). Flaviviruses are assembled using a dense and organized array of three structural proteins (Envelope, E, Membrane, prM, and capsid, C), the viral genomic RNA and a host lipid envelope (13). Flaviviruses entry into a target cell is dependent on the glycoprotein E binding to a cellular receptor(s) (13). It has been shown that flaviviruses use a wide range of cell surface receptors for entry into host cells, that includes C-type Lectin, AXL and MER, TYRO3 and α_V_β_3_ integrins (14). Flaviviruses enter host cells *via* clathrin-mediated endocytosis. During this process, a series of organizational and conformational changes in viral E glycoprotein occurs, that are triggered by low pH for consequent viral membrane fusion, and thus resulting in the release of nucleocapsid into the host cell cytoplasm (11, 13).

Recent research highlights have shown tetraspanins to play critical role(s) in immune response-based therapies towards flaviviruses (7). Although, the immunological mechanism is essential for the prevention, clearance and control of infection, but clinical challenges are usually linked with immunopathogenesis and virus-specific immunity (15). Therefore, there is an urgent demand for development of course effective and safe vaccine(s) and or novel approaches/methods requiring improved understanding of the immune response involved during viral infections. Currently, there are no therapies/specific drugs or vaccines available for many of the arthropod-borne flaviviral diseases. Despite substantial progress in understanding the role of tetraspanins in numerous diseases, the physiological and therapeutic importance of these family of proteins in arthropod-borne viral diseases remains largely unknown. In this review, we provide a broad overview on the role of tetraspanins in virus-host interactions, virus-mediated vesicular trafficking, crosstalk with extracellular vesicle (EVs) and exosome, in the regulation of immune response mechanism(s) and in their molecular interactions with RNA. We also discuss the prospects for considering tetraspanin(s) as novel therapeutic candidates to target arboviral diseases.

## Tetraspanins Organization and Their Role as Regulators of Virus-Host Interactions

**Figure 1:**
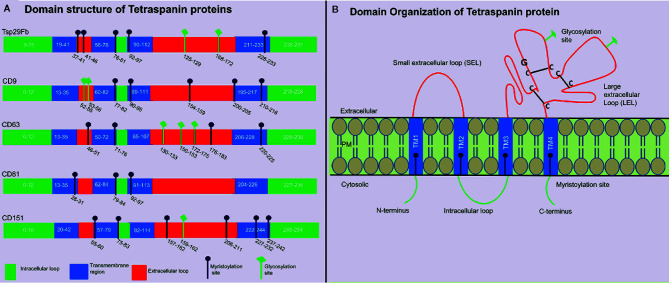
Tetraspanin structural organization and domain analysis from arthropod and vertebrate host. **(A)** Schematic diagram showing tetraspanin domain organization predicted at PROSITE (ExPASy) using primary amino acid sequence of mosquito *A. aegypti* Tsp29Fb (accession no: AAEL012532-RA), human- CD9 (accession no: NP_001760.1), CD63 (accession no: NP_001771.1), CD81 (accession no: NP_004347.1), and CD151 (accession no: NP_001034579.1). N-glycosylation sites are predicted using NetNGlycb (http://www.cbs.dtu.dk/services/NetNGlyc) and PROSITE (ExPASy). N-glycosylation sites, which were predicted in both web tools, are shown. Domain analysis demonstrates presence of conserved tetraspanin domain, 4 transmembrane regions, and variable number of glycosylation/myristoylation sites, and amino acid sequences. Different regions in tetraspanin proteins are indicated with different colors and labels. **(B)** Schematic representation of several characteristic features in tetraspanin protein is shown in the context of its transmembrane architecture. Tetraspanins have four highly conserved transmembrane domains (TM1-4), two extracellular portions known as small extracellular loop (EC1/SEL) and large extracellular loop (EC2/LEL), one intracellular loop (trans-passing from domain 2-3), and N- and C-terminal tails. EC2/LEL domain is conserved among several tetraspanins with 2–4 disulphide bonds (indicated with black lines) formed between cysteine residues in CCG-motif. These residues are binding sites for many interacting proteins and a strong epitope region for anti-tetraspanin antibodies. Numerous tetraspanins show glycosylation (shown in green lines) in the extracellular loops and palmitoylation sites (shown in blue line) at the intracellular border of the four transmembrane domains.

Tetraspanins are recognized to play significant role(s) in the pathology of infectious diseases caused by dengue, corona, hepatitis and influenza viruses (3). Tetraspanins are an evolutionarily conserved superfamily of transmembrane domain-containing proteins with 37 members in *Drosophila melanogaster*, and 33 members in humans with characteristic features and orthologs in other distantly related species, such as insects and arthropods (16). The evolution and origin of tetraspanins is largely known but most of these family member’s functions is less studied or remained unknown due to the difficulties to understand their subtle functions and/or to identify their functional redundancies (17). Overall, homology is highly conserved between isoforms in their primary amino acid sequence except at the small variable domain located within the large extracellular loop (LEL). These variations in the LEL region could explain the functional differences between isoforms, if they exist within the class of individual members (18). Structural analysis of different tetraspanins identified a common pattern of four transmembrane domains in all tetraspanins, but they vary in number of amino-acid residues, glycosylation pattern and myristoylation sites (19). Glycosylation and myristoylation sites in *Aedes aegypti* Tsp29Fb, human- CD9, CD63, CD81 and CD151 are predicted using NetNGlyc (http://www.cbs.dtu.dk/services/NetNGlyc) and PROSITE (ExPASy) and are shown in (**Figure 1A**). These small integral membrane proteins with four transmembrane segments (TM1-4), also have two extracellular loops of unequal sizes (SEL and LEL), one short intracellular turn/loop and the N- and C-terminal intracellular tails (**Figure 1B**) (4). Tetraspanins can be differentiated from other protein families based on their particular structure of second LEL. Tetraspanin proteins have 200–300 amino acids, and in addition they possess characteristic disulfide bonds, a cysteine–cysteine–glycine (CCG) motif in the large outer loop, membrane-proximal palmitoylation sites, and at least 2-4 hydrophilic cysteine residues within transmembrane domains (20, 21). Tetraspanin molecule has also been reported to possess one or more potential glycosylation, myristoylation, and lipid modification sites that participates in the regulation of signal transduction, proteolytic processing, budding, viral entry and replication, and in viral-host interactions (22). Viruses such as WNV do not have their own glycosylation machinery (23). However, it employs the host cell processes to glycosylate its proteins in order to invade and release from the host cell (23). Recent studies have shown the crystal structure of CD81 (24) and CD9 (25), which exposes a reversed cone-like architecture, suggesting the key mechanistic details for modulation of tetraspanin function. Tetraspanin enriched micro-domains (TEMS), also known as tetraspanin web, a distinct class of membrane domains, share the ability to associate with different transmembrane receptors (26). TEMs can contain specific clusters of lipids, transmembrane proteins, membrane associated proteins, and even signaling molecules (26). They have been implicated in regulation of tetraspanins function such as lymphocyte activation, as adhesion receptors involved in inflammation, pathogen infection and are proposed as potential candidates for development of novel therapeutic approaches (26, 27).

### Role of Mammalian Tetraspanins in Virus-Host Interactions

Among all the cell surface proteins, Cluster of Differentiation (CDs such as CD9, CD63, CD81, CD151, CD82) and Tetraspanin (Tspan) types that includes Tspan7 and Tspan9 have been reported to be involved in viral infections (1). Studies investigating different viral structures have shown that specific tetraspanin proteins are selectively linked with particular virus and have functions in various stages of infectivity, ranging from attachment to syncytium formation and release from host cells (2, 3). Tetraspanins play multifunctional roles in viral entry and exit process (2). Viruses modulate and use a particular tetraspanin molecule as compartmentalizing host entry factor or as a receptor or co-receptor (1). Coronavirus and Influenza A-viruses are enveloped RNA viruses (28). TEMS, specially containing CD9 and CD81 molecules, are the best entry sites for both these viruses as they are functionally required for viral fusion (29). Several co-receptors have been identified that are used by these viruses for glycoprotein-catalyzed process (2, 30). CD9 facilitated condensation of proteases and receptor permitted rapid and efficient entry of MERS-CoV virus into host cells (30). CD81-positive endosomes are necessary for the membrane fusion and entry of influenza virus (29). Coronavirus membrane fusion is facilitated by the viral spike glycoprotein (S) and depends on numerous processes such as conformational changes of the S protein and proteolytic processing (28). Experiments with mice and cell lines lacking CD9 revealed that this molecule is critical for entry of human coronavirus strain 229E (28). Likewise, CD81 is involved in HCV entry through direct interaction with the viral glycoprotein E2 that acts as a virus receptor (31). Tetraspanins enhance HCV binding by activating EGFR signaling pathways, which allows tetraspanin/receptor complex-assembly formation and stimulates CD81-CLDN1 or CD81-EGFR complex formation (32). Numerous proteins that contribute in the early steps of HCV infection are recognized to be located in tetraspanin-interaction network or in those tight junctions. For example, CD81, a member of tetraspanin network, is shown as one of the important components in predicting proteins necessary for early steps in HCV infection (33). CD81 acts as receptor that directly bind with envelope glycoproteins E1 and E2, thus, facilitating entry of HCV and viral binding to host cells (33). Moreover, CD81 interaction with glycoprotein E2 has been shown to participate in viral binding and recognition (34). Several of the DNA viruses have been also shown to modulate the effects of mammalian tetraspanins. The human herpesvirus (HHV)-6A receptor CD46 is known to form complexes with the tetraspanin CD9. Using a genetic approach, knockouts of CD9 in SupT1 cells had reduced expression of immediate early transcripts but deficiency for CD46 showed abolished binding and reduced infection in SupT1 cells. This data indicated a negative role of CD9 for CD46-independent infection. These studies also suggested that HHV-6A is strictly dependent on CD46 for entry, although other proteins, like CD9, may enhance the infection in host cells (35). Benayas and colleagues showed that the loss of CD81 on herpes simplex virus (HSV) type-1 infected cells compromised replication of viral DNA and formation of new infectious particles (36). Overall, these evidences suggest that viruses use tetraspanin molecules, proteases, and clusters of receptors for their entry and trafficking in mammalian host cells.

### Arthropod Tetraspanins in Virus-Host Interactions

**Figure 2:**
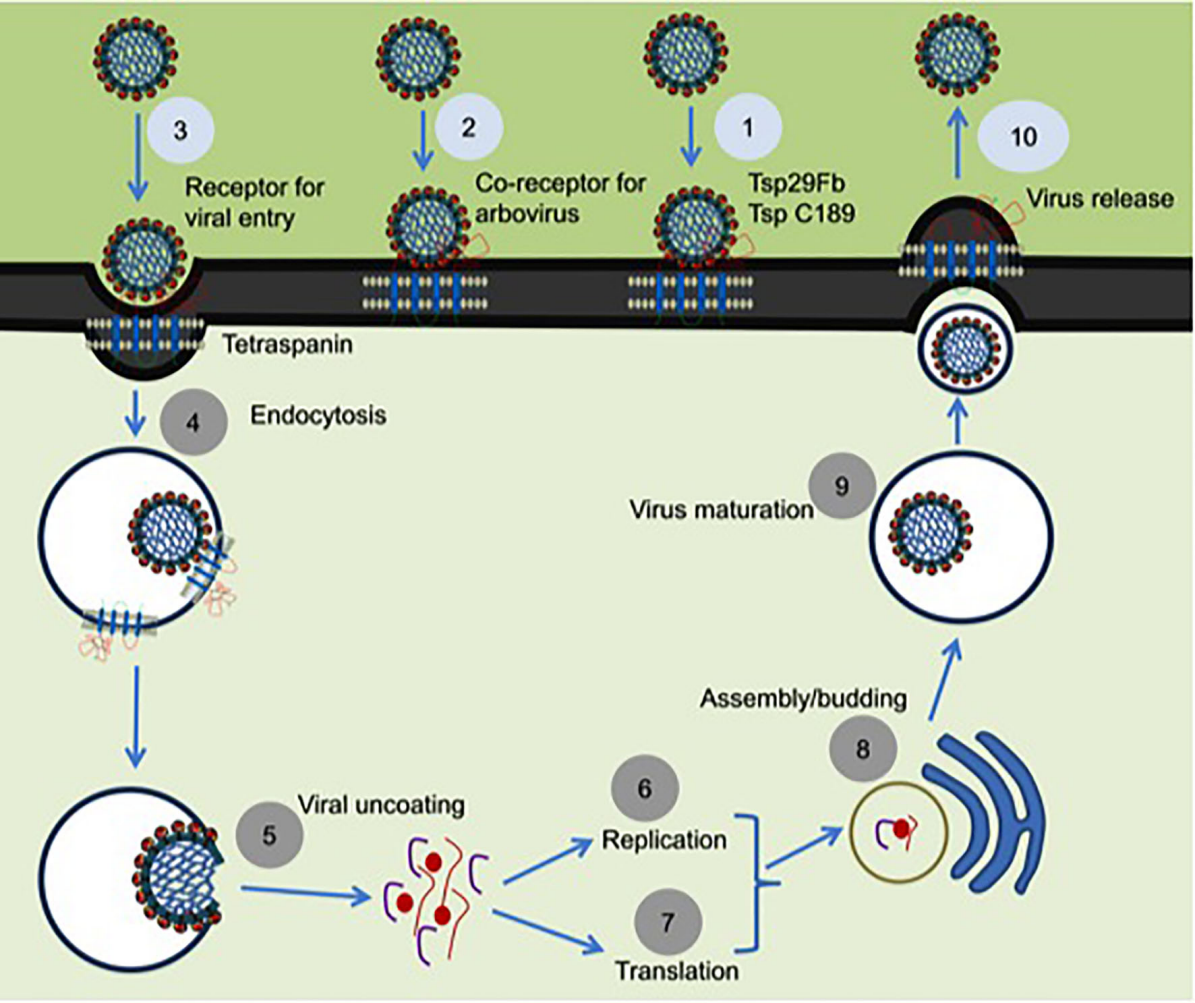
Proposed model for the role of tetraspanins in viral interactions. Mosquito tetraspanin Tsp29Fb is involved in DENV2/3 transmission from arthropod to mammalian cells, and Tsp C189 play a key role in cell-to-cell spreading of viral particles (indicated as step 1). Tetraspanin Tsp29Fb directly interacts with viral E-protein and perhaps might act as co-receptor for viral entry (denoted as step 2), and in specific serve as a receptor in case of arbovirus entry during clathrin-dependent receptor-mediated endocytosis (shown as step 3) and/or for internalization of viral particles by endocytosis (indicated as step 4). Tetraspanin proteins trigger the fusion and viral uncoating process (represented as step 5). Tetraspanins in association with several other host proteins are implicated in replication (denoted as step 6) and in translation process (shown as step 7). Tetraspanins participates to enable the delivery of viral genomes into the nucleus for successful infection and assembly processes (noted as steps 8). Tetraspanin contribution is highlighted in budding and assembly formation (noted as step 9) and in membrane fusion events can be exploited for viral exit from cells *via* direct release of secretory vesicles such as exosomes (represented as step 10). The step numbers have been classified into two color codes (light blue or dark gray). Role of arthropod tetraspanin in entry/exit of flaviviruses is proposed research and indicated in light blue color, whereas the published data is represented as dark gray circles.

Arthropods comprises the largest phylum on earth and have impacted human health significantly throughout the evolutionary history (37). As disease vectors, arthropods have substantial interactions with humanity (38). Arthropod vectors play significant role(s) in the transmission of numerous infectious agents in tropical and subtropical regions around the globe (39). Arthropods have established utmost strategies to oblige as successful vectors for pathogen transmission due to their ability in biting host, taking blood meal from hosts and allowing microbial replication and survival in vertebrate host for an extensive period of time (40). Mosquitoes are the primary medically important vectors that transmit several of the arboviruses including dengue. Dengue fever in humans is a painful, devastating mosquito-borne disease caused by DENV with more than 50 million cases per year, worldwide (41). DENV exists as four serotypes (DENV1-4) that causes dengue hemorrhagic fever (DHF), multiple organ failure, and death in humans (41). The relationship of arthropod tetraspanins with viruses seems to be predominantly complex. Our study has shown that arthropod tetraspanin domain containing glycoprotein, Tsp29Fb, mediates transmission of DENV2/3 from mosquito to mammalian cells (6). Immunoprecipitation analysis showed a direct co-association and interaction of Tsp29Fb with DENV2 E-protein, thus indicating an important function of tetraspanin in facilitating transmission and replication of DENV2 (6). Silencing of *tsp29Fb* expression resulted in a substantial reduction in DENV2 loads in cells and significantly decreased export of the viral E-protein and RNA genome in exosomes (6). These findings evidently demonstrated the importance of arthropod tetraspanins in facilitating DENV2 release, replication, and transmission (6). Overall, our study suggested that inhibition of arthropod tetraspanin Tsp29Fb or inhibition of EVs *via* GW4869 (a cell permeable, selective inhibitor for neutral sphingomyelinase (N-SMase)) are both potential therapeutics to block DENV2/3 and perhaps other mosquito-borne flaviviruses (6). A mosquito tetraspanin C189 has been found to be upregulated ~4-fold upon DENV2 infection (42). This tetraspanin was revealed to co-localize with viral proteins particularly in the plasma membrane of infected cells and in the intracellular membranes (**Figure 2**) (42). This study suggested that due to their involvement in intercellular adhesion, tetraspanins are presumably essential for the spread of DENV2 from cell-to-cell (42). In addition, tetraspanin C189 is crucially involved in cell-to-cell spreading of DENV particles in C6/36 cells (7). It has been shown that RNAi mediated knockdown of tetraspanin C189 in mosquito cells reduced virus transmission from cell-to-cell (7). However, complementation with C189 expression restored cell-to-cell transmission of DENV in mosquito cells. This study further observed increased cell-to-cell transmission of DENV at the site where the donor cell directly interacts with the recipient cell (7). A recent study has shown that tetraspanin C189 containing vacuoles mediate cell-to-cell spread of viral RNA within DENV2-infected C6/36 cells (See **Table 1**) (43). Magnetic immune isolation (MI) and sucrose gradient centrifugation showed that DENV2 RNA is transmitted by C189-VCs from donor to recipient cells. Viral RNA was only detected in recipient cells in transwell system, indicating that cell-cell contact is essential for DENV2 intercellular spread (43). C189-VCs served as carriers for transmission of viral RNA and virions to neighboring cells after established contact, suggesting persistent infection in mosquito cells and well-organized dissemination of DENV2-infection within mosquito vector (43). Delineating the role of arthropod tetraspanins is an interesting avenue to investigate the mechanisms of viral transmission.

**Table 1:** Tetraspanin functions in various cellular processes.

Tetraspanin	Pathogen	Involvement in biological function/process	Reference
**Arthropod tetraspanins**			
Tsp29Fb	DENV2	Transmission of DENV2/3 from arthropods to mammalian cells	(6)
Tetraspanin C189	DENV2	Cell-to-cell spreading of DENV2	(7, 42)
	DENV2	C189-containg vacuoles participate in transmission of viral RNA and virions to neighboring cells	(43)
**Host tetraspanin**			
CD81	HCV	Direct interaction with the viral glycoprotein E2 and acts as a virus receptor	(31, 33)
	HCV	Participate in viral trafficking	(34)
	HCV	CD81 partner EWI-2 inhibit viral entry	(44)
	HCV	Role for extracellular vesicles in securing the virions	(45)
		Modulate B cell receptor-mediated signaling	(46)
		Ag processing and presentation	(47)
	IAV	Viral uncoating and budding	(29)
CD63	EBV	Involved in intracellular signaling	(48)
	HCV	Viral trafficking from late endosomes to lysosomes	(49)
CD9	MERS-CoV	Viral entry and fusion	(30)
CD151		TEM assembly	(50)

## Tetraspanins in Vesicular Trafficking and Regulation of EVs/Exosomes Upon Viral Infection

**Figure 3:**
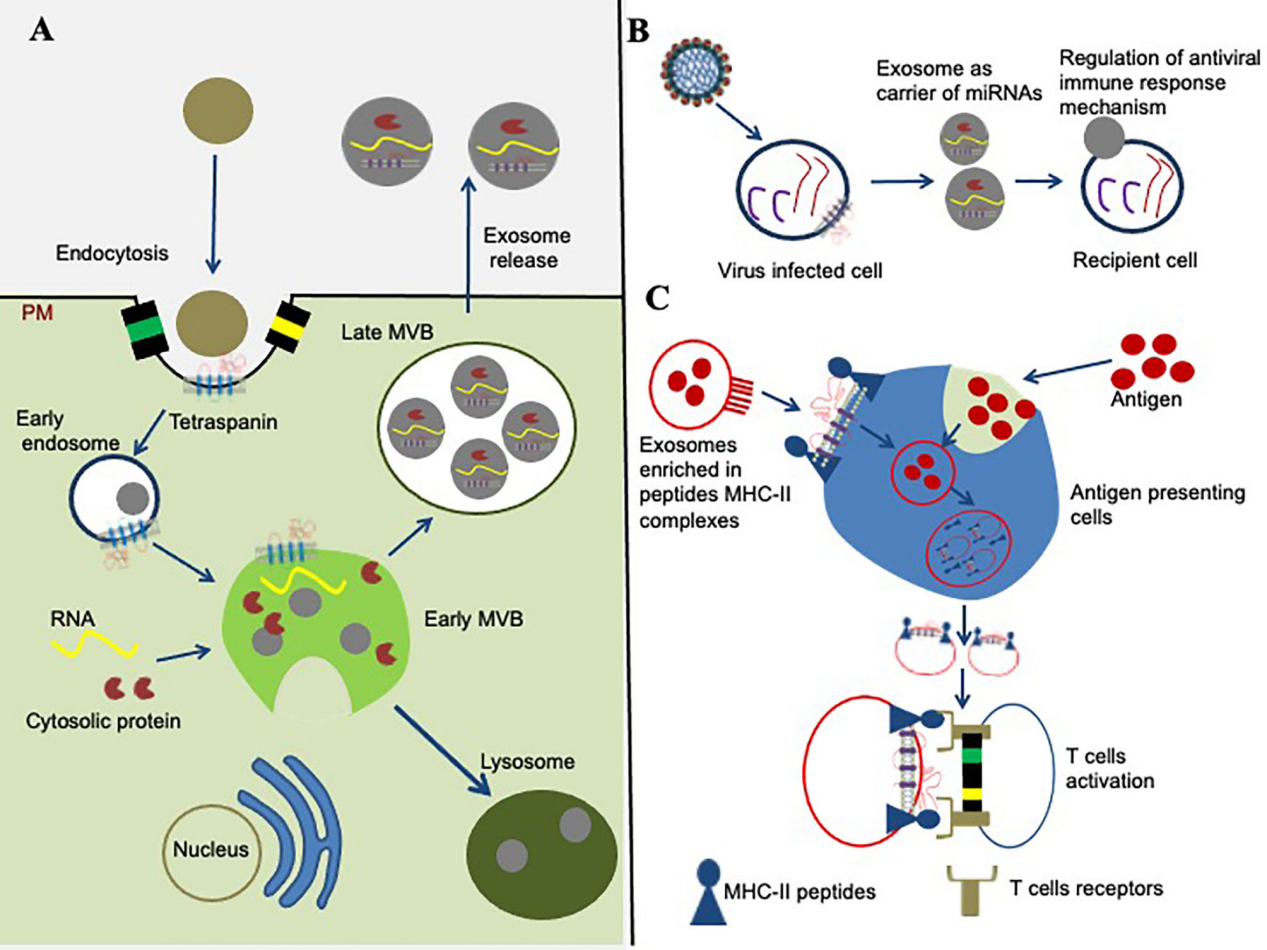
Role of tetraspanins in exosome biogenesis and modulation of immune signaling. **(A)** Role of tetraspanins in exosome biogenesis is shown. The early endosome formation is the first step in vesicle uptake and recycling. Development of early multivesicular bodies (MVBs, shown in green complex) is dependent on sorting of cytosolic proteins, clathrin-coated membranes, RNA molecules, tetraspanin-enriched domains (TEMs), and lipid rafts. The sorting and packaging of several other molecules into exosomes cannot be ruled out and has not been shown for simplicity. Some mature multivesicular bodies (MVBs) fuse with the hydrolytic lysosome, where the vesicular cargo is subsequently degraded. The membranes of late MVBs fuses with the plasma membrane resulting in the release of exosomes/EVs into the extracellular environment. **(B)** Tetraspanins role in miRNA modulation is shown. Tetraspanins directly or indirectly interacts with different signaling molecules and receptor(s) at the membranes/lipid rafts, and organize into the specialized tetraspanin-enriched micro-domains (TEMs) that might play significant role during exosome cargo sorting (miRNAs, RNA, and proteins) from viral-infected cells to recipient cell. **(C)** Tetraspanin involvement in signaling pathway is shown. Exosomes are potential in the context of modulation of an immune response through their ability to present MHC–peptide complexes to specific cells, resulting in activation of T cells and antigen presentation cells.

Tetraspanins and their TEMs received increased attention for their contribution to exosome biogenesis (**Figure 3A**), and as fundamental components that influence the function of exosomes in vesicular trafficking, internalization, and deviation from degradation in the proteasome (27, 51). Enrichment of tetraspanins in EVs/exosomes influences progressive consideration in unraveling protein-lipid and protein-protein interactions, intracellular vesicular sorting and membrane dynamics (27, 51). Exosomes have been defined as greatly enriched in tetraspanins (52, 53). Besides a set of cytosolic molecules and common membrane components, the human exosomes comprises of several tetraspanins including CD151, CD63, CD9, CD53, CD82, CD37, CD81, and Tspan8 and they are often used as exosome biomarkers (54). Exosomes are membrane-bound vesicles, typically of 40–120 nm in diameter that are released from most cell types such as immunocytes, blood cells, platelets and endothelial cells (53). Their biogenesis is constitutively generated by inward budding of early endosomes (53). Early endosomes then mature into late endosomes before formation of intraluminal vesicles (ILVs) within large multivesicular bodies (MVBs) (53–55). Tetraspanin and tetraspanin-associated proteins that are positioned by TEMs are arranged into small vesicles during the formation of MVBs (**Figure 3A**). Cytoplasmic protein(s) and RNA molecules can be internalized into MVBs *via* ceramide-dependent or ESCRTs pathways (55, 56). The molecules from the MVBs fuses with the plasma membrane resulting in the release of membrane-bound vesicles into extracellular space that ultimately becomes an EVs/exosomes, or the vesicles fuses with the hydrolytic lysosomes, where these components are subsequently degraded (55).

Exosomes have been reported to exploit viruses entry route for cargo delivery (54). Exosomes isolated from HCV-infected human hepatoma cell lines transmitted virions in the presence of neutralizing antibodies (45). Binding of tetraspanin CD81 to HCV established a complex that exploited the fusogenic capabilities. This HCV-CD81 complex was transferred as cargo to circulate in hepatocyte cell-derived infectious exosomes (45). This study indicated a possible role for EVs in securing the virions and facilitating viral pathogenesis (45). Additionally, Shrivastava and collegues have shown the role of tetraspanin CD63 with autophagy proteins in HCV particle secretion and release within exosomes. They also reported that exosomes carry infectious viral RNA fragments and tetraspanins play a key role in HCV trafficking from the late endosomes to lysosomes (49). Tetraspanins, CD81 and CD63 are both enriched on exosomes-derived from HCV-infected cells and CD81 has been shown to act as receptor for HCV E2 glycoprotein (57). Interferon-inducible transmembrane proteins (IFITM) have been reported as effector antiviral molecules that reduce the entry of flaviviruses, including impact on DENV-mediated cellular tropism that is independent of receptor expression. Exosomes carrying IFITM3, enables transmission of antiviral activities from cell to cell upon DENV infection (58). The molecular mechanism(s) employed by arthropod-borne flaviviruses to bypass the blood–brain barrier (BBB) is an unclear and poorly understood topic. However, it is reasonable to hypothesize that exosome-mediated crossing of the BBB might be a crucial mechanism for flavivirus neuropathogenesis (59). Our current studies are highly focused on undertaking these ideas to reveal these mechanisms. Recent study revealed that genome of ZIKV could be identified in the fetal brain and the amniotic fluid, which endorses that exosome pathways contribute in the viral transfer across the placental barrier (60). Likewise, in *in vitro* conditions, neuronal exosomes aid in ZIKV transmission between murine cortical neurons in embryonic brains (61). It was reported that neuronal exosomes contain both ZIKV RNA and proteins that modulate infection of this virus to naïve recipient cells (61). Furthermore, assays performed with neutralizing antibodies and RNaseA-treatments revealed that ZIKV proteins/RNA were present inside of the exosomes (61). Exosomes serve as functional carriers of mRNAs and miRNAs between cells, suggesting their role in cell–cell communication (62).

Exosomes secreted from DENV-infected cells mediate safe viral transmission, thereby, enabling virus protection from anti-dengue neutralizing antibodies (59, 63). Our recent reports provided evidence for the first time to suggests that flaviviruses use exosomes for their transmission from arthropod to vertebrate cells through interaction(s) with arthropod exosome-enriched proteins (6, 53, 64). A molecular and cellular study reported a novel role for Tsp29Fb, an increased tetraspanin in arthropod exosomes, in mosquito-dengue virus interactions (6). Another study reported that exosomes-derived from DENV-infected *Aedes albopictus* C6/36 cells contained a protein that showed cross-reactivity to an antibody against the human CD9 (65). Exosomes released from DENV-infected C6/36 cells were bigger in size than the ones secreted from uninfected cells (65). These exosomes were able to infect naïve C6/36 cells, indicating that exosomes play a significant function in DENV dissemination (65). It was noted that arthropod exosomes play key roles in transmission of flaviviral RNA and proteins to naïve human cells (64). This study not only provided evidence that transmission of LGTV is facilitated by arthropod-derived exosomes but also that LGTV uses exosomes for dissemination to the vertebrate host cells (64). Both negative and positive LGTV RNA strands, viral envelope (E) and non-structural (NS1) proteins, and perhaps polyprotein were identified in exosomes-derived from tick and murine host cells (64). Treatment with GW4869, not only reduced exosome production and release but also affected LGTV loads in exosomes-derived from tick and neuronal cells (64). Another recent study revealed that LGTV-mediated suppression of *Is*SMase plays a key role in exosome biogenesis and in membrane-associated viral replication, which provided new insights in the involvement of vector defense mechanism(s) and antiviral pathways against tick-borne viral infections (66). Our novel discovery showing the presence of *in vivo* exosomes in tick saliva and salivary glands revealed a role for these arthropod EVs in regulating the immune response at the tick-human skin interface *via* IL-8 and CXCL12 (67). Treatment of human skin keratinocytes (HaCaT cells) with tick saliva/salivary gland-derived exosomes induced IL-8 and reduced CXCL12 chemokine that lead to dramatic delay in cell migration, wound healing and repair process during the scratch assay (67). Exogenous treatment of CXCL12 (2 µg of purified protein) completely restored these delays and indeed enhanced the repair process suggesting a skin barrier protection role for this chemokine at the tick bite site (67). Overall, this study showed that tick salivary exosomes secreted in saliva regulates host immune response at the tick bite site to allow continuous blood flow and persistent feeding. We believe that tick or mosquito encoded antigens would be ideal candidates for the development of blocking the transmission of vector-borne flaviviruses.

## Tetraspanins Modulate Immune Responses and Related Mechanism(s)

The tetraspanins and their associated-proteins propose possible models for molecular interactions in modulation of the immune system (68). Tetraspanins are expressed on all cells of immune system, which provides a scaffold that enables the temporal and spatial engagement of their associated proteins (1, 68, 69). Tetraspanin are expressed on leukocyte subsets such as macrophages, monocytes, T and B lymphocytes, dendritic cells, granulocytes and natural killer cells (68–70). These proteins bind directly or indirectly with different molecules -for example, B-cell receptors, T-cell receptors, α4β1 and α6β1 integrins, major histocompatibility complex (MHC) class I, MHC class II etc., that play significant role(s) in the modulation of different immune cells (69, 71). Enrichment of tetraspanins on exosomal membranes suggests a novel mode in intercellular communication, which has significant function in numerous cellular processes, including antigen presentation, signal transduction, and modulation of immune responses (1, 68, 69). Evidences on the role of CD81 in immune cells has now begun to deliver concrete links between particular function and a specific molecule (70). These include roles associated with CD4 in T cells, integrins in B cells and T cells, CD81 with CD19 and MHC class II molecules in B cells (**Figure 3B**) (69). Crosslinking of tetraspanins CD9 and CD81 antibodies modulated T cells, suggesting their function as positive effectors (72). Furthermore, CD151, CD37, CD81, and TSPAN32 down modulated T-cell triggering in response to *in vitro* stimulation by sequestering key molecules into TEMs (73). CD81 also contribute to efficient association and molecular organization between the B cell receptor and its partners (72).

Studies have shown the involvement of tetraspanin connections with MHC class I and class II, including MHC’s on T cells (CD82), and antigen-presenting cells (CD9, CD81) as both of them distribute into the immune synapses (**Figure 3C**) (74). Tetraspanin CD151, TSPAN32, and CD63 have been shown to be associated for normal platelet functions. Platelets from tetraspanin CD63-null mice showed delayed clot retraction, impaired spreading on fibrinogen and defective platelet aggregation (75). CD81 is the most studied tetraspanin on immune-cells, and its significance has now been recognized in different functions including Ag-induced B cell activation, as a receptor for HCV entry, T-cell activation, Ag processing and presentation (47). Moreover, CD81-enriched microdomains has been shown to organize Nano-clusters to modulate B cell receptor-mediated signaling (46). Tetraspanin CD63 functions as a bridge in endosomal and autophagic processes to modulate the major viral oncoprotein. It affects the latent membrane protein 1 (LMP1) levels for exosomal secretion and allows intracellular signaling of Epstein-Barr Virus (EBV) within infected cells (48). It has been demonstrated that CD63 is essential for efficiently packaging of LMP1 into exosomes/EVs (48). During EBV infection, CD63 act as a negative regulator and directly opposes intracellular signaling activation downstream of LMP1 including mTOR cascade to deactivate host cell autophagy and to facilitate cell growth and proliferation (48). Interactions of tetraspanins with pattern-recognition receptors (PRRs) have provided novel insights into the organization of Ag receptors in modulation of downstream signaling pathways (76). The C-type Lectins receptors (LARs) are promising candidates for recognizing pathogen associated molecular patterns (PAMPs), and direct targeting of these LARs is commonly used as effective strategy to treat flaviviral infections (77). Tetraspanins collaborates with C-type Lectins to enhance viral attachment to cells and thus facilitates flavivirus infection (78). Collectively, these evidences highlight the new insights in the involvement of tetraspanins to modulate immune responses.

## Putative Roles for Noncoding RNAs in Modulation of Tetraspanins

Noncoding RNAs (ncRNAs) molecules play a variety of functions such as siRNAs has been involved in antiviral defenses, miRNAs and piRNAs controls transposons and regulation of gene expression, and lncRNAs mainly implicated in modulation of immune response mechanism of flaviviruses (79). Exosomes generated from HCV-infected cells induces the myeloid-derived suppressor cells through direct targeting of the miR-124-mediated signaling pathway (57). The miRNAs-125b and -146a targeting tetraspanin-12 enhances the NF-κB activity, suggesting that these miRNAs have tremendous potential to modulate innate immune response in brain cells (80). Recent study has shown the important role of exosomes as vehicles for miRNAs (including miR-223, let-7a, miR-150, miR-1229, miR-23a, miR-1246, and miR-21) transport to recipient cells (81). Studies on miRNAs targeting exosomal tetraspanin such as CD151, CD9 and CD81, suggests that exosomes are present in all bodily fluids, and that exosomes serves as diagnostic markers and therapeutic delivery vehicles for infectious diseases (82).

Flaviviral infections accumulate different patterns of ncRNAs relevant in immune evasion and viral pathogenesis that differently regulate viral fitness in host. A key characteristic feature of this advancement is recognition of the importance of ncRNAs interaction(s) with RNA-binding proteins (83). RNA-binding proteins has been known to play key roles in miRNAs mediated gene regulation, regulation of miRNA biogenesis, and in recognition and modulation of miRNA targets (83). A recent study has shown to increase incorporation of miRNAs in exosomes that regulate gene expression involved in pro-inflammatory and antiviral responses upon WNV infection (84). Next-generation sequencing approach has identified a potential role of *Ixodes scapularis* tick miRNAs in viral replication including POWV (85), and LGTV (86). Our group has also recently shown that repression of tick miRNA facilitates rickettsial pathogen survival and transmission to the vertebrate host (87). Currently, microRNA-targeting has been developed as an active strategy for pathogenicity and for the selective control of tissue-tropism of RNA viruses (88). In addition, small RNA profiling reveals differential expression of host piRNAs and miRNAs that modulates DENV2 replication in *A. aegypti* mosquito cells (89). A study has reported differential expression of arthropod miRNAs (that are highly conserved in vertebrates) upon infection of mosquitoes with flaviviruses such as ZIKV, DENV and WNV (90). These findings indicate an important role for miRNAs in targeting tetraspanins that could serve as potential therapeutic targets.

Viruses have established numerous strategies to enhance the efficacy of replication and to encounter antiviral immune system. Among these strategies adopted by viruses, lncRNA molecules are involved in viral-vector interaction(s) and have greater impact on viral biology (91). Arthropod-borne flaviviruses produce sub-genomic flavivirus RNAs (also known as sfRNAs that are ∼300-500 bases in length) or Xrn1–resistant RNAs (xrRNAs) (92). Both lncRNAs have a mutual molecular mechanism for their generation, but they have differences in various functions implicated in the flavivirus life cycle (93). Besides this, sfRNA play a significant role in RNAi-mediated pathway that impact in modulating antiviral immune response (94). Another study has shown that substitutions in the NS5 (non-structural 5) protein increases the delivery of sfRNA to new susceptible cells in order to inhibit type I IFN induction, which might be used as an effective strategy to control DENV replication (95). Silencing of tetraspanins (CD151, CD81, CD63 and CD9) expression in A549 cells resulted in significantly lower sfRNA copies in exosomes, indicating that sfRNA is packaged in infectious particles containing DENV envelope (E) protein or in virions (95). CD63 antibodies were used to deplete exosomes to determine whether sfRNA is packaged exclusively in virions or in exosomes. Although, exosomes could be removed from cell culture supernatant, but they could not be fully depleted, and sfRNA:gRNA ratios remained higher in culture supernatant, indicating that sfRNA is securely contained in DENV E-protein containing particles including exosomes or in virions (95). Several evidences have exposed the involvement of sfRNA in targeting host-derived proteins upon flavivirus infection (96, 97). Genome wide profiling of lncRNA expression identified *MAGED1*, *STAT1*, and *IL12A* genes that might play a key role as biomarkers for designing new strategies to prevent DHF (91). Transcriptomic analysis of lncRNAs profile of *A. aegypti* cells identified a set of differentially expressed immune-related genes implicated in IMD and MAPK signaling pathway responsive to DENV infection (98). Collectively, these observations highlight the significance of ncRNA in viral replication, transmission, and immunity, but further research enhancement on this topic is needed to fully reveal the mechanism(s). Future studies should focus not only to understand the involvement of ncRNAs in regulation of the vertebrate host immune response but also should emphasize on delineating the role of ncRNA in vector-pathogen interactions.

## Tetraspanins as Therapeutic Targets Against Flaviviral Infections

DENV, ZIKV, and WNV infect millions of people worldwide. Tetraspanins could be considered as strong therapeutic target(s) due to their role in viral interactions. Strategies such as RNA interference (RNAi) and antibody-mediated blocking of tetraspanin proteins function could be developed as ideal approach to prevent viral infections. Treatment with antiviral drugs such as viral-cell receptor interaction inhibitors, interferon’s, and viral enzyme inhibitors could lead into significant reduction in the viral loads but these may not completely eradicate viruses (99). Additional difficulties associated with these approaches include low efficacy owing to viral genotype specificity, toxicity and side effects, development of viral resistance, patient immune variability and high cost (99). Progress to develop novel vaccines for flaviviruses has been hindered by poor capacity and potential pathogenicity to elicit protective immune responses (99). Tetraspanins can be broadly classified into two groups based on their function of direct interactions with viral-expressed proteins, and their dependence on TEMs that effects host-cell functions. Tetraspanin–pathogen direct interactions reflect a necessity of the invading pathogen to interact with TEMs (1). Numerous viruses are assumed to have established complex interactions to ‘highjack’ or exploit lipid rafts (3). TEMs may provide many viruses with ‘gateways’ to enter and leave host cells without any interference (1, 3). Models where tetraspanins directly interact with viral proteins provide better understanding for the development of therapeutic strategies. These strategies should focus to specifically block viral replication, prevent mutations in the viral genome and cause no adverse effects to the host (99). Advance use of such approaches is of great concern and these methods are becoming progressively more prominent with the failure of traditional methodologies of vaccination that were used to control and treat viral infections (99). Also, the appearance of new strains due to mutations in viral genome in human populations set limits for these traditional methodologies (100). The best characterized example of tetraspanin specific direct interaction is CD81 with HCV E2 glycoprotein that implies the small-molecule inhibitors targeting this tetraspanin could prove valuable treatment for HCV infection (101). A study demonstrated that CD81’s large extracellular loop (LEL) had no effect on infection by serum-derived HCV, whereas CD81 down regulation by siRNA or anti-CD81 antibodies markedly inhibited the entry process of HCV (102). Tetraspanin CD81 plays a critical role in HCV infection *in vitro*, and anti-CD81 antibody administration have been shown to exhibit a protective role *in vivo* (103). The immunoglobulin superfamily member IgSF8 (also known as EWI-2) directly binds to tetraspanin CD81. Therefore, inhibiting the interaction between HCV E2 glycoprotein and CD81, will result in the blockade of viral entry (44).

The palmitoylation of tetraspanin CD9 and CD151 plays a significant role in TEM assembly that could possibly suggests novel target for microbial infections (50). It has been shown that palmitoylation of CD9 plays a role in mediating DHHC2 suppression in A431 cells to affect cell performance (50). CD151 palmitoylation has been reported to play significant function in assembly of the large network of cell surface tetraspanin-protein interactions, distribution in tetra-CD151 showed markedly diminished stability during biosynthesis. Disruption of tetraspanin domain through palmitoylation dynamics may therefore represent a candidate therapeutic strategy to combat viral infections (104). The siRNA-mediated knockdown of tetraspanin CD151 or antibody-mediated blocking of CD81 was shown to reduce virus entry/internalization. This data suggested that CD81 and CD151 could possibly be targets for blocking HCV infection (105).

Genome wide RNAi-based screens are used for identification of host proteins that play a significant role in advancement of clinically important data (106). A set of host proteins involved in the regulation of cellular pathways for many aspects of WNV infection have been identified (107). Using the same RNAi approach used for WNV, it has been revealed that approximately half of the factors are unknown (that may contain mosquito TSPs) and one third of the known factors were involved in the replication of DENV2 (108). Interestingly, silencing of gene expression of proteins that were up-regulated upon WNV infection also affected DENV2 infection, demonstrating that these proteins play a vital role in restriction of flaviviruses growth (108). Among the differentially expressed proteins, 116 genes were considered important as insect host factors for DENV2 propagation, vesicular trafficking (like VATPases and accessory proteins), and among which 82 of the host factors had identifiable human homologs (108). The siRNAs targeting human homologs revealed that 42 of these proteins considered as positive factors that affected DENV2 growth, showed notable conservation of required factors between insect, vector and the mammalian hosts (108). In addition, antibodies against specific tetraspanin’s that are exogenously internalized could be delivered *via* recombinant exosomes (109). Arthropod CD63 ortholog Tsp29Fb has been recently described as a target for this type of therapies in viral infection. In our previous study, we revealed that arthropod EV-enriched tetraspanin domain-containing glycoprotein, Tsp29Fb play an important role in transmission of DENV2 (6). Identification of this tetraspanin in vector host, has conceivably suggested Tsp29Fb as potential biomarker for future therapeutic approaches to combat arthropod-borne viruses. Recent advances in genetic engineering technologies have made it possible to create mosquitoes that block key interactions between the mosquitoes and pathogens, this includes physically blocking the key interactions required for the pathogen development. It has been shown that genetically engineered *A*. *aegypti* mosquitoes efficiently expressed a DENV-targeting single-chain variable fragment (scFv) derived from a previously characterized broadly neutralizing human antibody, which blocked the infection and transmission in these mosquitoes (110). Taken together, all these studies provide evidence that targeting tetraspanins may provide novel strategies to inhibit critical processes, and aid in the development of potential therapeutic targets to treat and/or control several of the vector-borne flaviviral infections.

## Conclusion and Future Perspectives

Arthropod-borne viruses are important pathogens that cause devastating diseases in humans. Development of transmission-blocking vaccines targeting vector or pathogen molecules is an effective strategy to eliminate and control arthropod vectors and to prevent arthropod-borne infections (111). Although, continuing efforts to improve and advance traditional approaches to fight against vector-borne diseases have not been ignored, however, novel strategies are required immediately. Recent evidence suggests that pathogens uses or associates with tetraspanins as a mean for entry, replication, and exit from the host cell. Our studies have shown that tetraspanin domain containing glycoprotein Tsp29Fb, an ortholog of human CD63 mediates transmission of DENV2/3 from arthropod to mammalian cells (6). Tetraspanin might play significant role(s) as receptors, as mediators in the compartmentalization of host entry factors at the membrane, as facilitators in fusion and cell-to-cell spreading of viral particles, and as activators of EGFR signaling pathways. Exosomal tetraspanin CD9, CD63 and CD81 are important molecules that are involved in many functions including vesicular trafficking, internalization of antigens and in transmission of human pathogens including DENV, WNV, ZIKV, LGTV and HCV. Tetraspanins are expressed on all immune cells providing a scaffold that enables the temporal and spatial engagement of their associated proteins. Tetraspanin proteins might bind directly or indirectly with different molecules such as, B-cell or T-cell receptors, α4β1 and α6β1 integrins, MHC classes I and II components in order to modulate the activity of immune cells during viral infections.

Tetraspanins also play a key role in the regulation of different cellular processes. RNAi-mediated repression of tetraspanin gene expression or antibody-mediated blocking of tetraspanins function could be employed as effective antiviral strategies to control infections. One of the other thoughts is will it be helpful to make knockout mosquitoes for different arthropod TSPs, and will it be feasible to free knockout or genetically modified mosquitoes lacking TSPs in the field. However, it is important to first delineate if these knockout mosquitoes will survive in nature without TSPs. Genetics and transgenetic are quite strong in mosquitoes when compared to ticks, however, there could be several challenges to make knockout mosquitoes. RNAi-mediated silencing of mosquito Tsp29Fb (a human ortholog of CD63) significantly reduced DENV2 loads in both mosquitoes and the infectious EVs derived from these cells. We did not observe any morphological or viability issues with the *A. aegypti* mosquito cells. This data indicates the possibility of generating feasible knockouts for Tsp29Fb and perhaps other TSPs in mosquitoes. Our immediate future line of research is centered on these important thoughts. Due to their important roles in several processes as discussed in this review article, therapeutic strategies based on targeting tetraspanins would provide better success for treating/preventing vector-borne diseases. Although, involvement of tetraspanin superfamily has been studied in different aspects, limited information is available for these molecules compared to the studies that reported on other transmembrane domain containing proteins, such as GPCRs and integrins. There are numerous questions that need to be answered with regard to the involvement of arthropod tetraspanin(s) to understand their full potential for therapeutics chassis a) how do arthropod tetraspanin(s) can be used as therapeutic targets for vaccine or antiviral drugs? b) How do ncRNAs interfere with tetraspanins in the modulation of immune responses and respective cellular mechanisms? c) How do three-dimensional structures of different tetraspanins appear? d) Could arthropod tetraspanins affect immune responses to flaviviruses and other microbes? e) What other putative viral co-receptors participate with arthropod tetraspanins to modulate the viral entry and release? f) Is it feasible to generate genetically modified vector such as mosquitoes or ticks lacking tetraspanin? In summary, detailed characterization of arthropod tetraspanin(s) will not only facilitate our understanding of pathogenicity of flaviviruses but also will lead to the development of novel effective therapeutic tools such as antiviral drugs and vaccines to combat vector-borne diseases.

## Author Contributions

All authors listed have made a substantial, direct, and intellectual contribution to the work, and approved it for publication.

## Funding

Research in this area is supported by R01 AI141790 grant award to HS from National Institute of Health (NIH)/National Institute of Allergy and Infectious Diseases (NIAID).

## Conflict of Interest

The authors declare that the research was conducted in the absence of any commercial or financial relationships that could be construed as a potential conflict of interest.
